# Exploring Attention Bias Mechanisms in Sub-Threshold Depression: ERP Insights into Biased Orientation and Disengagement

**DOI:** 10.3390/bs14090821

**Published:** 2024-09-14

**Authors:** Xin Zhang, Huibin Jia, Enguo Wang

**Affiliations:** Faculty of Education, Henan University, Kaifeng 475000, China; 18003780841@163.com (X.Z.); huibin_jia@foxmail.com (H.J.)

**Keywords:** inhibition of return (IOR), attention orientation, attention disengagement, event-related potentials (ERPs), sub-threshold depression (SD)

## Abstract

Individuals with depression may have alterations in attention that begin at the sub-threshold stage. This study explored attention bias from the perspectives of early attention orientation and late attention disengagement in individuals with sub-threshold depression (SD) and healthy control (HC) individuals using a cue-target paradigm and event-related potentials (ERPs). The study enrolled 46 participants, comprising 23 males and 23 females, with 25 individuals in the SD group and 21 in the HC group, exceeding the calculated sample size requirement. The data were analyzed from two aspects. Behavioral data showed that SD individuals had difficulty in attention disengagement and that the time of attention transfer was delayed. Analysis of ERP data revealed that, regardless of the attributes of the emotional stimulus, the cue information promoted participants’ response to the target stimulus. While SD individuals did show directional acceleration of attention to the emotional stimulus, no significant negative attention bias was observed. Taken together, these findings suggest that SD individuals do not show specific directional acceleration of attention to negative stimuli in the early stage of attention processing, although there may be attention avoidance.

## 1. Introduction

Major depressive disorder (MDD), recognized as a profoundly disabling psychiatric condition, presents itself through its defining features of sustained melancholy and diminished capacity to experience pleasure, complemented by secondary symptoms like fluctuations in body weight, disrupted sleep patterns, or feelings of listlessness [1]. MDD has a high incidence rate, a long duration, a high recurrence rate, and is associated with a high suicide rate [2]. The first national epidemiological survey of mental disorders in China reported that the lifetime prevalence of MDD is 3.4% and that lifetime disability exceeds 47% [3].

Numerous studies indicate that progression from healthy development to MDD is continuous [4], with sub-threshold depression (SD) existing as a key middle stage. A person exhibiting select symptoms of depression, such as a diminished interest in activities or a state of low mood, who, however, does not fulfill the necessary diagnostic thresholds for MDD due to the symptoms being either too few, not severe enough, or not lasting long enough, is identified as having SD [5,6]. SD is also called sub-syndromal depression, sub-clinical depression, minor depression, mild depression, and limited depression [7,8]. Although the symptoms are less severe than MDD, they can significantly reduce an individual’s quality of life and impose a significant financial burden [9,10].

The incidence of SD is significantly higher than that of MDD [11]. People diagnosed with SD face a significantly elevated risk, approximately five times greater, of progressing to MDD in comparison to those who are considered healthy [12]. Thus, SD is considered an early stage of MDD and is of important predictive value for MDD [13]. Early intervention has a significant effect on SD and can effectively prevent progression to MDD [14]. Consequently, a deeper comprehension of SD can significantly improve our insight into the onset and progression of MDD [15] and provide a scientific basis for interventions for individuals at high risk of depression.

Attention is the first step in the cognitive process and the primary link for processing external stimuli [16]. Attention bias refers to the allocation of attention resources. Previous research indicates that people exhibit varied patterns of attention allocation towards emotional stimuli in contrast to neutral ones [17]. According to the component theory of attention, attention is not a single structure but includes sub-processes such as attention orientation, attention disengagement, and attention avoidance [18]. In line with this perspective, attention bias comprises three components [19], namely, facilitated attention, difficulty in attention disengagement, and attention avoidance.

The stage at which attention bias arises is controversial. Some studies have found that impaired attention orientation can explain avoidance of negative information in individuals experiencing the early stage of depression [20]. In contrast, other studies suggest that attention bias in individuals with MDD is not characterized by a rapid shift to negative stimuli, but by the fact that once attention is captured by negative stimuli it is difficult to disengage [21,22]. In other words, negative information may directly interfere with MDD individuals’ attention disengagement function, causing them to pay excessive attention to negative information and affecting detection of targets. It is thus impairment of the attention disengagement function for negative information that leads to a stronger negative attention bias in MDD [23]. Besides these two hypotheses, it has been suggested that attention bias may relate to early attention orientation and late attention disengagement. Using a spatial cue task, Koster et al. [24] demonstrated that individuals with MDD showed both accelerated attention orientation and attention disengagement difficulties when the cue acquired a threat value through aversive conditioning and when the cue presentation time was 200 ms.

In the process of information processing, attention orientation occurs in the early automatic stage while attention disengagement and attention avoidance occur in the late strategic stage, with the former stage regulated by the amygdala and the latter regulated by higher cortical areas [19,25]. As prior studies suggest that early acceleration of attention orientation and late attention disengagement difficulty are important to the formation and maintenance of mood disorders [19], this study zeroes in on the initial orientation of attention and the subsequent disengagement phase of attention bias in individuals diagnosed with SD.

Building on foundational work in affective neuroscience and research into cognitive biases associated with depressive disorders, our study seeks to contribute to the existing literature by examining attention bias in individuals with SD. Recognizing the pivotal role of early attention processes in the development and persistence of depressive symptoms, we hypothesize as follows:

**Hypothesis** **1.** *Early Attention Orientation—Individuals with SD will demonstrate an accelerated orientation towards emotional stimuli in the early stages of attention processing compared to healthy controls (HC), suggesting an enhanced sensitivity to emotional information.*

**Hypothesis** **2.** *Late Attention Disengagement—Despite initial rapid orientation, individuals with SD will encounter difficulties disengaging their attention from emotional stimuli, resulting in an extended focus on these stimuli.*

By testing these hypotheses, our study aims to offer a more nuanced understanding of attention processes during the early phases of depressive symptomatology. The insights gained could inform the development of early intervention strategies that target attention biases, with the goal of preventing or reducing the risk of progression to major depressive disorder.

## 2. Materials and Methods

### 2.1. Participants

G* POWER 3.1 software was utilized to determine the necessary sample size for the study. Based on an effect size of 0.25 (the effect size of 0.25 was estimated based on preliminary data from a pilot study and was used to calculate the required sample size for detecting a medium effect with adequate power), a desired statistical power level of 0.95, and a significance level (α) of 0.05, the analysis suggested that 28 participants would be sufficient. Consequently, the study enrolled 46 participants, comprising 23 males and 23 females, with 25 in the SD group and 21 in the HC group, exceeding the calculated sample size requirement. After enrolling 46 participants (23 in each group), we conducted a post hoc power analysis using G*Power to calculate the actual power of the study. With the observed effect sizes in reaction times and ERP components, the actual power of our study is calculated to be 0.88, indicating a high probability of detecting significant effects if they exist.

Drawing from prior research, participants in this study were grouped using a secondary screening method [26]. A total of 308 university students from Henan University underwent initial screening using the Beck Depression Inventory-II (BDI-II). Only fully completed surveys were taken into account. Subsequently, those with scores ranging from ≤6 to ≥14 were extended an invitation to take part in the study within a week after the initial assessment. Prior to the study, participants filled out the Self-rating Depression Scale (SDS) and underwent an interview conducted by a psychology graduate student proficient in the DSM-V criteria [27] to confirm the absence of MDD and any associated conditions. Participants with BDI-II scores of ≤2 and SDS scores below 53 were categorized into the HC group, whereas those with BDI-II scores of ≥14 and SDS scores at or above 53 were placed in the SD group. Since even minor co-occurring conditions could impact ERP measurements, a subsequent assessment was performed on all participants using the Trait Anxiety Inventory (TA-I) component of the State-Trait Anxiety Inventory (STAI). The findings indicated no statistically significant disparity in T-AI scores between the SD and HC groups (*p* > 0.05).

The criteria for participation were specific: Participants were required to be right-handed, possess either normal vision or vision that could be corrected to normal, be free from color vision deficiencies, and have no severe physical ailments. They were also required to have no recent history of acute or chronic infections within the past fortnight, to have abstained from psychotropic medications or antipyretic analgesics for the same period, and to have not used immunomodulatory drugs or hormonal treatments for a minimum of six months. Furthermore, exclusionary factors included a lack of past incidents involving trauma, inflammatory conditions, fever episodes, allergic reactions, or substance misuse involving alcohol or drugs.

This study was approved by the Henan province key laboratory of psychology and behavior. Prior to commencing the experiment, the participants were briefed on the study’s particulars. Each participant then offered their written consent to participate and received compensation upon the study’s completion.

### 2.2. Experimental Task and Stimuli

The cue-target paradigm is a commonly used experimental paradigm in attention bias research. In this paradigm, the cues appear in one of two boxes on the screen and the target stimulus appears at random. Participants were instructed to promptly assess the position of the target stimulus and to respond accordingly [28]. When the target stimulus and cue stimulus appear on the same side they are “effective cues”, whereas when they appear on the opposite side they are “ineffective cues”. If the response time (RT) to the target stimulus is shorter when the cue is an emotional stimulus, this indicates that participants have an attention bias towards the emotional stimulus.

**Figure 1:**
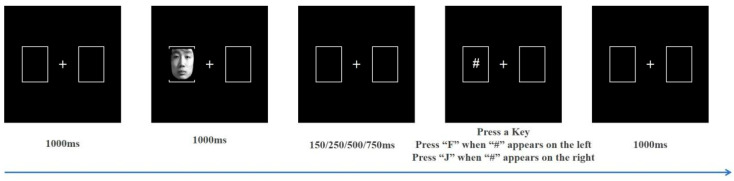
Stages of the cue-target task.

This study selected 30 images each of positive (happy), neutral, and negative (sad to angry in a 1:1 ratio) emotional facial expressions from the Chinese Affective Face Picture System (CAFPS) to serve as cue stimuli [29], with an equal distribution of male and female faces. As shown in Figure 1, the screen features a pair of boxes positioned on its left and right sides. The white fixation point in the middle of the two boxes automatically disappears after 1000 ms, and one of the boxes will then contain the cue stimulus (i.e., emotional face). The cue stimulus will automatically disappear after 1000 ms. The target stimulus “#” appears after stimulus onset asynchrony (SOA) of 150 ms, 250 ms, 500 ms, or 750 ms. Participants must judge the “#” position as soon as possible and respond accordingly, press the “F” key in response to the “#” symbol appearing on the left side of the screen, and the “J” key when the “#” symbol is on the right side. A blank screen then appears for 1000 ms, after which the next trial begins. Participants completed a total of 360 trials, organized as 60 trials for each of the six conditions, namely, positive emotion—effective cues, positive emotion—ineffective cues, neutral emotion—effective cues, neutral emotion—ineffective cues, negative emotion—effective cues, and negative emotion—ineffective cues (hereinafter referred to as positive—effective, positive—ineffective, neutral—effective, neutral—ineffective, negative—effective, and negative—ineffective). The cue and target stimuli were randomly displayed on the left and right sides of the screen in a 1:1 ratio.

### 2.3. Electroencephalogram (EEG) Recording

EEG recordings were made using a Quick Amp EEG system from Brain Products, Gilching, Germany, equipped with 64 Ag/AgCl electrodes mounted on an ActiCap, positioned following the International 10–20 system guidelines, with reference to the FCz electrode. Eye movements were tracked with bipolar electrodes for electrooculogram (EOG) measurements. Electrode impedances were maintained under 5 KΩ. The data underwent a DC-280 Hz band-pass filter during online acquisition, with a sampling frequency set at 1000 Hz.

The EEG data were processed offline using Analyzer 2.2, a software designed for the analysis of electrophysiological data, and the resulting figures were prepared using the same software suite. The offline processing of EEG data was based on the following series of steps: the reference electrode was replaced with the bilateral mastoids (TP9, TP10); a low-cut filter at 0.3 Hz, a high-cut filter at 30 Hz, and a 50 Hz notch filter were applied to all channels; independent component analysis (ICA) was utilized to eliminate EOG artifacts; and “Fp2” was designated for vertical EOG correction to mitigate the influence of blinking or eye movements. After the removal of artifacts due to equipment or participant actions, data were segmented from −200 to 1000 ms and baseline corrected from −200 to 0 ms. Participants with more than 50% trial rejections were excluded from the analysis [30]. The data were then subjected to superposition and averaging across six conditions: “positive—effective”, “positive—ineffective”, “neutral—effective”, “neutral—ineffective”, “negative—effective”, and “negative—ineffective”. The average amplitude of each ERP was superimposed with at least 30 trials.

### 2.4. Statistical Analysis

Data were analyzed using a 2 (Group: SD, HC) * 4 (SOA: 150 ms, 250 ms, 500 ms, 750 ms) * 3 (cue emotion: positive, neutral, negative) * 2 (cue type: effective, ineffective) analysis of variance (ANOVA). In this ANOVA, the group was considered a factor that varies between subjects, whereas SOA, cue emotion, and cue type were treated as factors that vary within subjects. Data with error or more than three standard deviations above or below the mean response were discarded.

For the behavioral data, RT and inhibition of return (IOR) were the dependent variables. IOR reflects inhibition control and was calculated by subtracting the average RT of effective cues from ineffective cues. If the average RT of effective cues is longer than that of ineffective cues, then the IOR effect exists [31,32]. For the EEG data, the P1 component is an early sensory response that occurs after stimulus onset, reflecting the initial visual processing of stimuli. Electrodes O1 and O2, located over the occipital area, are traditionally used to measure the P1 component because they are close to the primary visual cortex, where initial visual information is processed. The amplitude of the P1 component is known to be modulated by attention and the emotional content of the stimuli, making these electrodes suitable for examining early attention biases towards emotional faces [33,34]. Therefore, the P1 component was detected within a 130–170 ms time window in the occipital region (O1, O2). The N170 is a face-sensitive component, indicating more complex processing related to facial identity and expression. Electrodes PO7 and PO8, located in the occipito-temporal region, are strategically positioned to capture the N170 component, which is believed to be generated in the fusiform gyrus, a brain area implicated in face perception [35,36]. These electrodes are chosen because the N170 amplitude is sensitive to facial expressions and can reveal how emotional faces are processed differently from non-face stimuli. This is crucial for understanding attention biases in depression [37,38]. Consequently, the N170 component was detected within a 170–220 ms time window in the occipito-temporal region (PO7, PO8). A repeated-measures ANOVA was employed to examine the behavioral and ERP data, contrasting the two participant groups—SD and HC—within the framework of variables such as cue emotion and cue type. Adjustments to the degrees of freedom for the F-ratios were made using the Greenhouse–Geisser correction method, and the Bonferroni correction was applied for handling multiple comparisons.

## 3. Results

### 3.1. Behavioral Data

#### 3.1.1. Participant Characteristics

Table 1 presents the demographic details and questionnaire results of the participants. No significant differences were observed between the groups regarding age, gender, or T-AI scores. However, the SD group exhibited significantly elevated scores on both the BDI-II and SDS, with *p*-values well below 0.001.

#### 3.1.2. RT

Table 2 displays the RT and IOR data of the participants. The repeated measures ANOVA results showed that the main effect of SOA was significant (*F*_(1,44)_ = 92.056, *p* < 0.001, *η*^2^ = 0.677). Further simple effect analysis showed that RT was shortest when SOA was 750 m, and that the difference was statistically significant compared to SOA of 150 ms, 250 ms, or 500 ms (all *p* < 0.001). RT was also significantly shorter when SOA was 500 ms compared to when SOA was 150 ms or 250 ms (both *p* < 0.001). When SOA was 250 ms, RT was significantly shorter than that when SOA was 150 ms (*p* = 0.001).

A significant interaction effect was observed between the SOA and the type of cue (*F*_(1,44)_ = 2.877, *p* = 0.049, *η*^2^ = 0.061), as well as between the group and the cue type (*F*_(1,44)_ = 4.805, *p* = 0.034, *η*^2^ = 0.098). Further simple effect analysis showed that, when SOA was 150 ms, the neutral—effective RT in the HC group was significantly shorter (*p* = 0.015) than the neutral—ineffective RT, indicating a cue effect. When the SOA was 250 ms, the positive—effective RT was significantly shorter (*p* = 0.012) than the positive—ineffective RT, indicating a cue effect. When SOA was 250 ms, the effective cue RT in the HC group was significantly shorter (*p* = 0.015) than the RT for an ineffective cue, and the positive—effective RT was significantly shorter (*p* = 0.008) than the RT for a positive–ineffective cue, suggesting that a cue effect appeared. When the SOA was 500 ms, the RT of an effective cue was significantly shorter than that of an ineffective cue (*p* = 0.026). At an SOA of 750 ms, the RT for an effective cue in the SD group was significantly longer compared to an ineffective cue (*p* = 0.047), suggesting the emergence of a return inhibition effect.

#### 3.1.3. IOR

**Figure 2:**
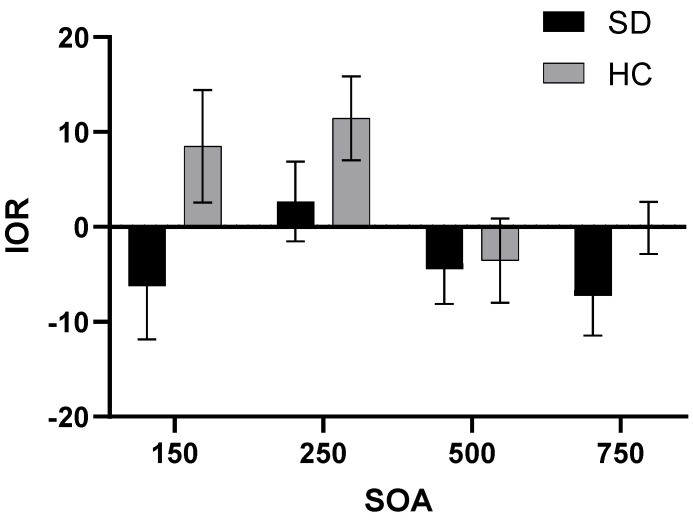
IOR for two groups under different SOA.

A repeated-measures ANOVA was conducted to assess the IOR, considering SOA and cue emotion as the variables that vary within subjects (Figure 2). The results indicated a significant main effect for the group factor (*F*_(1,44)_ = 4.805, *p* = 0.034, *η*^2^ = 0.098), with the SD group demonstrating a longer IOR than the HC group. Additionally, the main effect of SOA was also significant (*F*_(1,44)_ = 2.877, *p* = 0.049, *η*^2^ = 0.061). The IOR of SOA at 250 ms was significantly shorter than that of SOA at 500 ms or 750 ms (*p*_SOA=500ms_ = 0.023, *p*_SOA=750ms_ = 0.034, respectively). The IOR when SOA was 750 ms was shorter than that when SOA was 500 ms, indicating that the return inhibition effect was more obvious when SOA was 750 ms.

### 3.2. ERP Data

#### 3.2.1. P1

**Figure 3:**
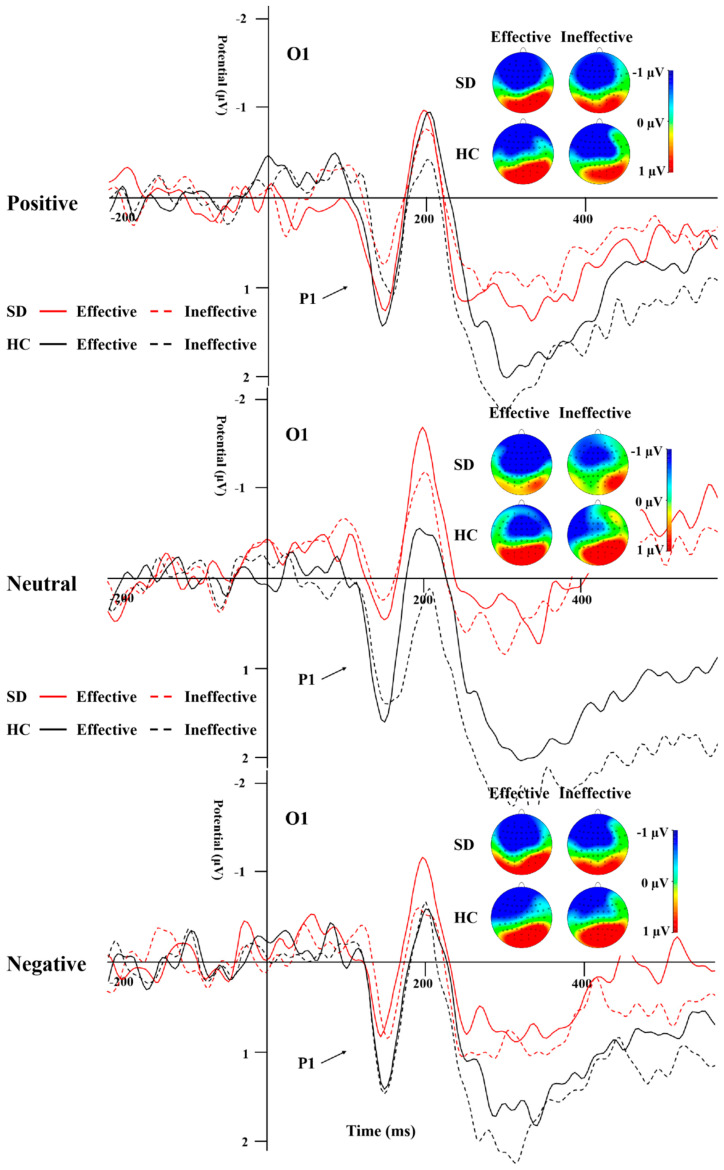
P1 elicited by the emotions and effective/ineffective conditions recorded at the O1 electrode.

Table 3 illustrates the peak amplitude and latency characteristics of the P1 component for both groups across all experimental conditions. A repeated-measures ANOVA was applied to the data from electrodes O1 and O2. The analysis revealed a significant main effect attributed to the group (Figure 3; *F*_(1,44)_ = 4.766, *p* = 0.034, *η*^2^ = 0.098), where the HC group exhibited a significantly more positive peak for P1 compared to the SD group. Additionally, the interaction effect between group and cue emotion reached significance (*F*_(1,44)_ = 3.94, *p* = 0.03, *η*^2^ = 0.082). Subsequent simple effect analyses indicated that the P1 peak was significantly more positive for neutral emotional cues in the HC group when contrasted with the SD group.

**Figure 4:**
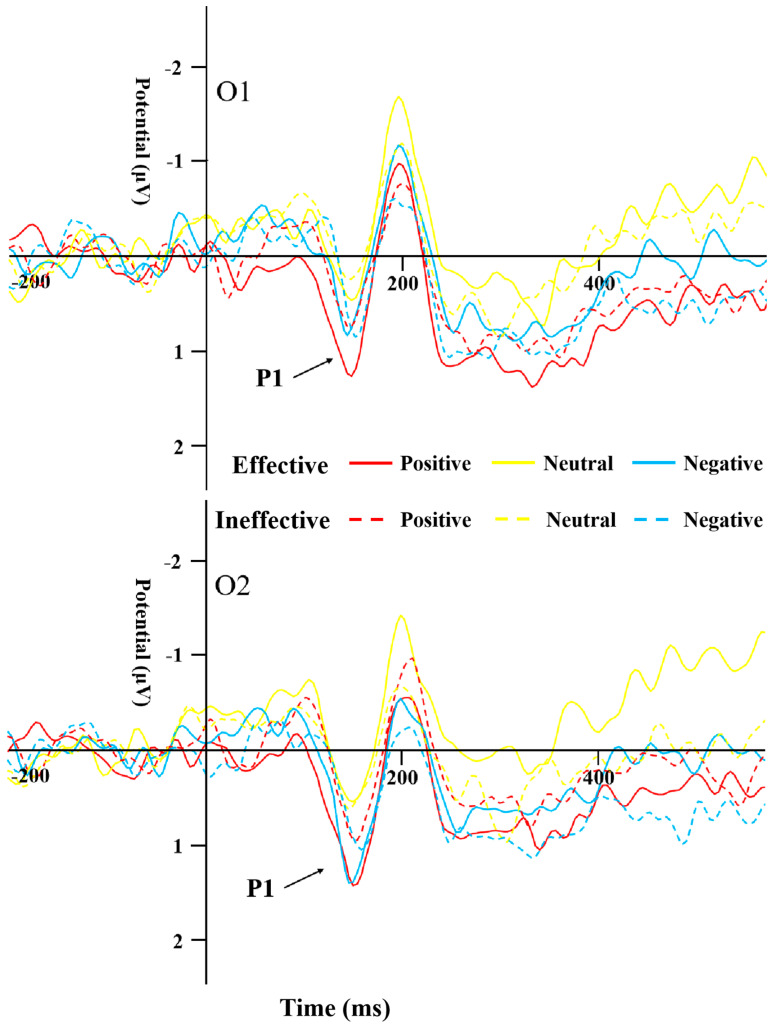
P1 induced by the different cue emotions and cue types in the SD group.

The results of the repeated-measures ANOVA on P1 latency indicated a significant main effect of cue emotion (*F*_(1,44)_ = 4.888, *p* = 0.01, *η*^2^ = 0.1), with the neutral emotional cues having a significantly shorter latency compared to the negative ones within the SD group (*F*_(1,44)_ = 4.295, *p* = 0.019, *η*^2^ = 0.152). Furthermore, the main effect of cue type was found to be significant as well (*F*_(1,44)_ = 6.158, *p* = 0.017, *η*^2^ = 0.123), and the latency of effective cues was significantly shorter than that of ineffective cues in the SD group (Figure 4).

#### 3.2.2. N170

**Figure 5:**
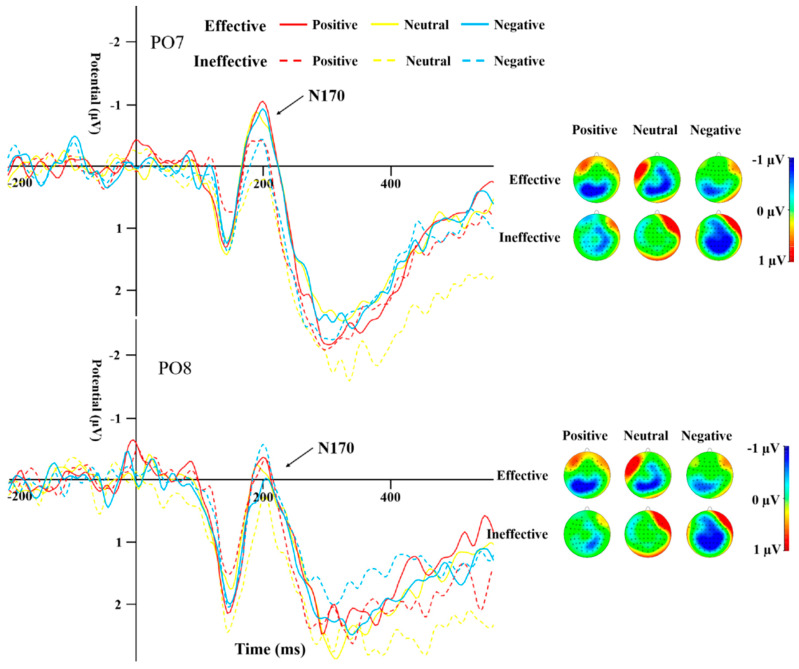
N170 induced by different cue emotions and cue types in the HC group.

**Figure 6:**
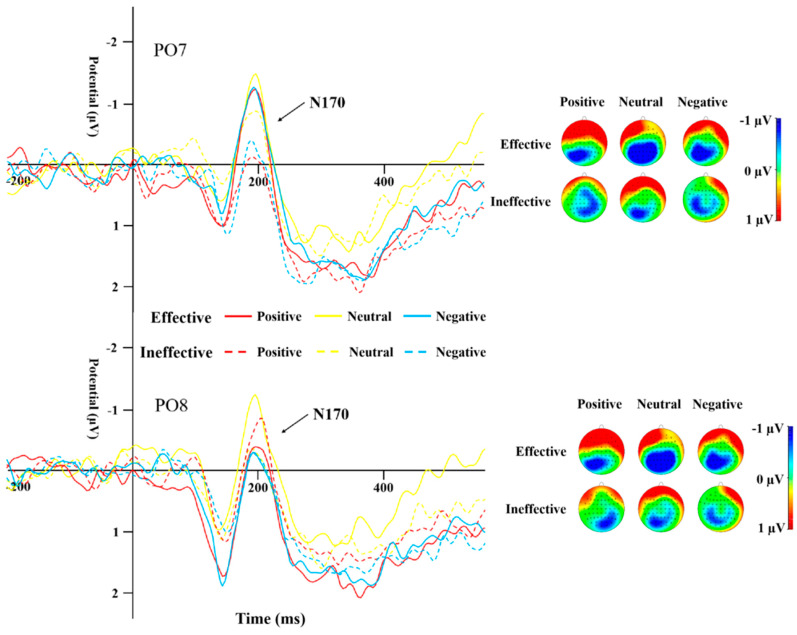
N170 induced by different cue emotions and cue types in the SD group.

Table 4 displays the peak amplitudes and latencies of the N170 component for both groups across various conditions. A repeated-measures ANOVA was conducted on the data from electrodes PO7 and PO8 for the N170. The results highlighted a significant main effect of cue type (*F*_(1,44)_ = 13.19, *p* = 0.001, *η*^2^ = 0.231). The N170 peak caused by ineffective cues was significantly more negative than that of effective cues (Figure 5 and Figure 6). The interaction effect between the type of cue and the side of the brain (hemisphere) proved to be statistically significant (*F*_(1,44)_ = 10.135, *p* = 0.003, *η*^2^ = 0.187), as was the interaction between cue emotion and cue type and hemisphere (*F*_(1,44)_ = 9.358, *p* < 0.001, *η*^2^ = 0.175). The interaction between group and cue emotion and cue type and hemisphere was also significant (*F*_(1,44)_ = 4.489, *p* = 0.017, *η*^2^ = 0.093). Subsequent analysis of simple effects revealed that under effective cues, the N170 peak in the left temporo-occipital area was notably more negative in comparison to the right temporo-occipital area. In the SD group, the N170 peak caused by effective cues was significantly more negative than that caused by ineffective cues in the left temporal-occipital region for positive and negative cue emotions, while the N170 peak caused by effective cues was significantly more negative than that caused by ineffective cues in the right temporal-occipital region for neutral cue emotions (Figure 6).

The repeated-measures ANOVA for N170 latency did not show any significant main effects or interactions.

## 4. Discussion

This study aimed to investigate the characteristics of attention bias and related ERPs in SD individuals from the perspective of early attention orientation and late attention disengagement. Analysis of behavioral data—mainly RT and IOR—indicated that the longer the SOA, the faster the participants reacted. When SOA was relatively short, the cue effect appeared in all cases, i.e., the response speed is faster for effective cues. These findings are consistent with previous studies [39], suggesting that cue information promotes responding to the target stimulus in the effective cues condition regardless of the attributes of emotional information, causing participants’ RT to the target stimulus to be shorter than that in the ineffective cues condition. Our results showed that when the SOA was 750 ms the effective cue RT in the SD group was longer than the ineffective cue RT, the return inhibition effect appeared, and the IOR was found to be significantly larger in the SD group compared to the HC group. As per existing research, the objective of return inhibition is to avert an individual’s focus from reverting to a previously attended location, thereby allowing attention to be redirected towards novel spatial areas [40,41], reflecting automatic and reflective removal of attention and realizing effective transfer of attention by removing (attenuating) input information so as to make efficient use of limited attention resources [42]. From our findings, it can be inferred that attention disengagement in SD individuals is difficult and that attention transfer is delayed compared to healthy individuals.

P1 and N170 are two early ERPs that have been extensively studied during early emotional face processing. The P1 of the occipital region is an important indicator of perceptual processing and is associated with activity in the striatal cortex [43]. The amplitude of this component is influenced by emotional factors [33] and escalates in response to the prominence of negative stimuli [34]. P1 is often used as an indicator of directed acceleration of attention [37,44]. In this study, overall, the P1 peak in the SD group was significantly less pronounced in positivity compared to the HC group, especially under the neutral emotion condition, suggesting that the overall response ability in SD individuals is weak. In the SD group, the P1 peak induced by positive and negative emotional stimuli was more positive than that induced by neutral stimuli, while the latency of P1 induced by positive and neutral emotional stimuli was shorter than that induced by negative emotional stimuli. In the HC group, the P1 peak and latency caused by the three emotional stimuli under the effective cue condition did not differ from those in the SD group. However, for ineffective cues, the peak P1 caused by positive and negative emotional stimuli was less positive than that caused by neutral stimuli, and the latency was shorter than that of neutral stimuli, indicating that SD individuals have early directional acceleration of attention to emotional stimuli compared to neutral stimuli, but no significant negative bias.

N170 mainly reflects cognitive processing of faces [35]. A study by Deldin et al. [38] found that individuals with MDD show less facial-specific N170 compared to their healthy peers. In the present study, the N170 peak for effective cues was significantly more negative than that for ineffective cues, indicating that cue information promoted the response to the target stimulus. Under the effective cues condition, regardless of the attribute of emotional information, participants’ executive control of the target stimulus was better than that under the ineffective cues condition. In the SD group, the peak for effective cues was significantly more negative than that for ineffective cues for positive and negative emotional stimuli; for the HC group, the peak difference between cue conditions occurred under neutral and negative emotional stimuli, i.e., the SD group showed stronger attention orientation to emotional stimuli. Unlike previous reports that N170 amplitude caused by negative stimuli in MDD individuals is significantly lower than that caused by positive and neutral stimuli [45], the peak for neutral emotional stimuli in the SD group in the present study was significantly more negative than that for negative stimuli, whereas there was no such difference in the HC group. This result suggests that SD individuals have attention avoidance to negative stimuli. In the HC group, the latency of ineffective cues was significantly shorter than that of effective cues under positive emotional stimulation, and no significant difference was found in the SD group. In other words, HC individuals showed accelerated orientation to positive information, while SD individuals did not.

The findings regarding attention disengagement and the lack of specific negative attention bias in SD are consistent with several recent studies. For instance, a meta-analysis by Bar-Haim et al. [17] supports the notion that attention biases in depression are more related to difficulty disengaging from negative stimuli rather than an initial orienting bias. Additionally, work by Gotlib and Joormann [22] emphasizes the role of cognitive factors, including attention, in the development and maintenance of depression. They suggest that once attention is captured by negative stimuli, it is hard to disengage, which aligns with our ERP data.

In summary, our findings indicate that individuals with SD show a general overreactivity to emotional stimuli, rather than a specific bias towards negative content. This suggests that early or pre-symptomatic stages of depression may involve a heightened sensitivity to the emotional environment. If this sensitivity predominantly encounters negative stimuli, it could contribute to the development of depressive disorders. Alternatively, this heightened reactivity might reflect an increased environmental sensitivity that could predispose individuals to depression when faced with ongoing negative environmental cues. These possibilities underscore the importance of considering the broader emotional landscape and its management in the context of early intervention and prevention strategies for depression.

## 5. Conclusions

This study used RT, IOR, and related ERP data to examine attention bias in SD and HC groups. The results of this study are generally consistent with those of previous work. Behavioral data showed that SD individuals have difficulty in attention disengagement and that the time of attention transfer is delayed. ERP data showed that, regardless of the attribute of the emotional stimulus, the cue information promoted an individual’s response to the target stimulus. SD individuals did show directional acceleration of attention to emotional stimulus, but no significant negative attention bias was found in SD individuals. However, it is worth noting that HC individuals showed directional acceleration of attention to positive stimuli, while no such attention bias was observed in SD individuals. Overall, these findings suggest there is no specific acceleration of attention orientation in response to negative stimuli in SD individuals at the early stage of attention processing, although there may be attention avoidance.

## Figures and Tables

**Table 1:** Basic information of participants and average score on screening scale (M ± SD).

	SD	HC	t	*p*
Male:Female	11:14	12:9		
Age	21.12 ± 2.297	22.38 ± 2.783	−1.684	0.099
BDI-Ⅱ	30.48 ± 6.035	1.76 ± 1.7	22.741	***
SDS	64.08 ± 7	32.54 ± 4.08	39.576	***
T-AI	31.28 ± 9.26	28.76 ± 4.5	1.201	0.238

*** *p* < 0.001.

**Table 2:** Averaged RT and IOR (in ms) for all trials in the HC and SD groups (M ± SD).

	SOA (ms)	Emotion	Effective	Ineffective	IOR
SD	150	Positive	442.49 ± 78.17	434.83 ± 87.71	−7.67 ± 42.43
		Neutral	430.95 ± 71.06	431.23 ± 77.10	0.28 ± 33.39
		Negative	437.78 ± 82.15	426.37 ± 80.77	−11.40 ± 38.96
	250	Positive	426.91 ± 80.32	432.96 ± 83.09	6.04 ± 31.84
		Neutral	425.95 ± 95.07	426.32 ± 79.19	0.36 ± 42.46
		Negative	425.65 ± 90.65	427.31 ± 84.00	1.66 ± 44.62
	500	Positive	407.34 ± 93.62	401.90 ± 86.07	−5.44 ± 48.31
		Neutral	385.13 ± 74.27	394.70 ± 83.48	−0.43 ± 30.63
		Negative	399.47 ± 77.01	391.95 ± 80.20	−7.52 ± 46.74
	750	Positive	384.64 ± 66.35	377.52 ± 61.19	−7.12 ± 26.43
		Neutral	391.47 ± 79.10	382.61 ± 65.02	−8.85 ± 39.71
		Negative	385.42 ± 67.78	379.68 ± 64.69	−5.74 ± 26.60
HC	150	Positive	429.16 ± 74.33	442.33 ± 62.65	13.17 ± 36.74
		Neutral	424.28 ± 57.40	443.78 ± 74.04	19.51 ± 37.71
		Negative	436.54 ± 75.03	429.41 ± 55.76	−7.14 ± 60.07
	250	Positive	399.61 ± 55.19	421.04 ± 67.69	21.43 ± 39.30
		Neutral	415.03 ± 67.17	425.21 ± 71.55	10.18 ± 40.47
		Negative	415.30 ± 66.09	418.06 ± 59.45	2.77 ± 36.99
	500	Positive	397.84 ± 61.82	387.98 ± 56.70	−9.86 ± 30.92
		Neutral	388.97 ± 65.59	387.16 ± 56.38	−1.80 ± 42.80
		Negative	391.96 ± 54.41	392.94 ± 54.55	0.98 ± 43.98
	750	Positive	388.34 ± 57.43	380.71 ± 60.19	−7.62 ± 27.91
		Neutral	382.23 ± 55.40	384.73 ± 68.42	2.50 ± 32.92
		Negative	374.24 ± 60.20	379.09 ± 55.44	4.85 ± 37.50

**Table 3:** The peak (μV) and latency (ms) of P1 in the both groups under all conditions (M ± SD).

			Positive		Neutral		Negative	
			Effective	Ineffective	Effective	Ineffective	Effective	Ineffective
Peak	SD	O1	1.51 ± 1.37	0.95 ± 1.42	0.74 ± 2.08	0.58 ± 1.65	1.15 ±1.81	1.0 ± 1.55
		O2	1.69 ± 1.32	1.19 ± 1.20	0.93 ± 2.21	0.95 ± 1.64	1.66 ± 1.31	1.36 ± 1.43
	HC	O1	1.70 ± 0.88	1.46 ± 1.49	1.88 ± 1.12	1.89 ± 1.11	1.63 ± 1.01	1.73 ± 1.22
		O2	1.89 ± 1.25	1.31 ± 1.10	1.78 ± 1.43	2.17 ± 1.39	1.98 ± 1.23	1.86 ± 1.23
latency	SD	O1	145.92 ± 10.34	149.60 ± 12.22	147.52 ± 12.18	149.12 ± 13.64	149.76 ± 11.78	152.00 ± 10.52
		O2	146.88 ± 11.58	151.52 ± 12.35	147.36 ± 13.15	148.48 ± 13.08	150.56 ± 10.45	156.32 ± 12.16
	HC	O1	149.33 ± 10.76	152.95 ± 11.52	148.95 ± 11.38	152.19 ± 12.55	152.00 ± 11.59	150.29 ± 10.85
		O2	150.48 ± 11.49	149.90 ± 11.29	148.57 ± 12.28	154.10 ± 8.26	154.67 ± 10.15	152.95 ± 9.03

**Table 4:** The peak (μV) and latency (ms) of N170 in both groups under all conditions (M ± SD).

			Positive		Neutral		Negative	
			Effective	Ineffective	Effective	Ineffective	Effective	Ineffective
Peak	SD	PO7	−1.59 ± 1.72	−0.64 ± 1.47	−1.73 ± 1.98	−1.40 ± 1.56	−1.62 ± 1.94	−0.86 ± 1.93
		PO8	−0.78 ± 1.80	−1.10 ± 1.53	−1.58 ± 2.55	−0.88 ± 1.35	−0.76 ± 1.96	−0.81 ± 1.83
	HC	PO7	−1.52 ± 1.40	−1.00 ± 1.72	−1.29 ± 1.76	−0.42 ± 1.97	−1.27 ± 1.61	−0.67 ± 1.22
		PO8	−0.96 ± 1.94	−0.77 ± 1.67	−0.74 ± 1.87	−0.03 ± 2.01	−0.47 ± 1.82	−0.81 ± 1.59
latency	SD	PO7	192.48 ± 13.73	192.00 ± 14.51	194.08 ± 13.62	194.40 ± 14.79	192.64 ± 13.84	194.40 ± 13.95
		PO8	196.80 ± 15.83	196.80 ± 13.76	194.72 ± 12.95	194.08 ± 13.32	197.28 ± 15.13	192.32 ± 15.01
	HC	PO7	197.71 ± 13.36	190.29 ± 13.95	194.48 ± 12.99	195.62 ± 15.79	195.43 ± 13.82	192.19 ± 13.53
		PO8	200.95 ± 13.38	193.33 ± 11.62	200.19 ± 13.65	196.95 ± 11.66	197.71 ± 15.26	200.00 ± 10.73

## Data Availability

The data that support the findings of this study are available from the corresponding author upon reasonable request.
